# The role of organizational context and individual nurse characteristics in explaining variation in use of information technologies in evidence based practice

**DOI:** 10.1186/1748-5908-7-122

**Published:** 2012-12-31

**Authors:** Diane Doran, Brian R Haynes, Carole A Estabrooks, André Kushniruk, Adam Dubrowski, Irmajean Bajnok, Linda McGillis Hall, Mingyang Li, Jennifer Carryer, Dawn Jedras, Yu Qing (Chris) Bai

**Affiliations:** 1 Lawrence S. Bloomberg Faculty of Nursing, University of Toronto, 155 College Street, Toronto, ON, M5S 3H4, Canada; 2 Department of Clinical Epidemiology and Biostatistics HSC-3V43C, McMaster University, 1200 Main Street W, Hamilton, ON, L8N 3Z5, Canada; 3 3rd floor CSB, Faculty of Nursing, University of Alberta, Edmonton, Alberta, T6G 2G3, Canada; 4 School of Health Information Science, University of Victoria, PO Box 3050 STN CSC, Victoria, BC, V8W 3P5, Canada; 5 SickKids Learning Institute; Department of Paediatrics, University of Toronto, 525 University Ave, Room 6021, Unit 600, Toronto, ON, M5G 2L3, Canada; 6 Registered Nurses’ Association of Ontario, 158 Pearl Street, Toronto, ON, M5H 1L3, Canada

**Keywords:** Health information technologies, Mobile technology, Personal digital assistant, Nursing informatics, Information-seeking, Organizational context, Evidence-based practice, PARIHS model

## Abstract

**Background:**

There is growing awareness of the role of information technology in evidence-based practice. The purpose of this study was to investigate the role of organizational context and nurse characteristics in explaining variation in nurses’ use of personal digital assistants (PDAs) and mobile Tablet PCs for accessing evidence-based information. The Promoting Action on Research Implementation in Health Services (PARIHS) model provided the framework for studying the impact of providing nurses with PDA-supported, evidence-based practice resources, and for studying the organizational, technological, and human resource variables that impact nurses’ use patterns.

**Methods:**

A survey design was used, involving baseline and follow-up questionnaires. The setting included 24 organizations representing three sectors: hospitals, long-term care (LTC) facilities, and community organizations (home care and public health). The sample consisted of 710 participants (response rate 58%) at Time 1, and 469 for whom both Time 1 and Time 2 follow-up data were obtained (response rate 66%). A hierarchical regression model (HLM) was used to evaluate the effect of predictors from all levels simultaneously.

**Results:**

The Chi square result indicated PDA users reported using their device more frequently than Tablet PC users (p = 0.001). Frequency of device use was explained by ‘breadth of device functions’ and PDA versus Tablet PC. Frequency of Best Practice Guideline use was explained by ‘willingness to implement research,’ ‘structural and electronic resources,’ ‘organizational slack time,’ ‘breadth of device functions’ (positive effects), and ‘slack staff’ (negative effect). Frequency of Nursing Plus database use was explained by ‘culture,’ ‘structural and electronic resources,’ and ‘breadth of device functions’ (positive effects), and ‘slack staff’ (negative). ‘Organizational culture’ (positive), ‘breadth of device functions’ (positive), and ‘slack staff ‘(negative) were associated with frequency of Lexi/PEPID drug dictionary use.

**Conclusion:**

Access to PDAs and Tablet PCs supported nurses’ self-reported use of information resources. Several of the organizational context variables and one individual nurse variable explained variation in the frequency of information resource use.

## Background

Healthcare professionals today have to manage an ever-increasing amount of clinical-related health information. To do so effectively and efficiently is crucial to the current context of healthcare delivery. An essential feature of information management is to ensure that the information is accessible at the times of decision making. In nursing, this utilization has been constrained by limited ability to access evidence-based guidelines at the point of care; however, many vehicles are currently available to provide more rapid and pro-active dissemination and implementation of best evidence. Information technologies, such as personal digital assistants (PDAs) and handheld computers (Tablet PCs), offer a solution for getting evidence to nurses directly at the point of care.

Evidence-based practice is the process by which nurses make clinical decisions using the best available research evidence, their clinical expertise, and patient preferences [1]. There is growing awareness of the role of information technology in evidence-based practice [2]. This awareness prompted the Ontario Ministry of Health and Long-Term Care (MOHLTC), Nursing Secretariat to create a funding opportunity to which healthcare organizations could apply to purchase personal digital assistants and mobile tablet PCs. Such devices were for use by frontline nurses to facilitate their access to evidence-based practice resources at the point of care. The MOHLTC ‘PDA initiative’ enabled access to three core electronic resources for mobile devices: drug and medical reference materials, best practice guidelines from the Registered Nurses’ Association of Ontario (RNAO), and Nursing Plus [3]. The drug and medical reference materials were either downloaded to the devices or available online and included an electronic drug and medical dictionary [4,5]. The RNAO launched the Nursing Best Practice Guidelines Program in 1999, with funding from the Ontario Ministry of Health and Long Term Care. The program is responsible for development, dissemination, implementation, and evaluation of nursing clinical and healthy work environment best practice guidelines. There are now 46 published guidelines, 38 of which are clinical best practice guidelines (BPGs) and eight that focus on healthy work environments. The guidelines were adapted for PDAs and smartphones at the time the Ministry of Health and Long-Term Care launched their PDA initiative, and most are available in Canada’s two national languages, English and French. They were available for online access.

Nursing Plus Best Evidence for Nursing Care from McMaster University's Health Information Research Unit allows nurses to register their areas of clinical interest to receive e-mail alerts about publication abstracts relevant to their interests. All citations (from over 140 clinical journals) are pre-rated for quality by research staff, then rated for clinical relevance and interest by at least three members of a worldwide panel of practicing nurses. Nurses have access to a searchable database of the best evidence from the medical literature, an e-mail alerting system, and links to selected evidence-based resources [ http://www.caretoknow.org/link/nursing-plus-best-evidence-nursing-care]. The Nursing Plus resources were available through online Internet access.

The Ontario Ministry of Health and Long-Term Care provided funding for the PDA initiative over two years. Organizations were able to apply for the funding on a competitive basis and those awarded funding in the first year were not eligible to apply for funding in the second year, thereby extending the initiative to a wider range of healthcare organizations over the two years. In a previously published paper [6], we reported the evaluation from the first year of the initiative. This paper reports findings from the second wave evaluation. In the first evaluation, we observed significant variation in nurses’ utilization of PDAs/Tablet PCs for information access across healthcare organizations and across healthcare sectors. Some of this variation was explained by differences among nurses (*e.g.*, ‘I am computer shy’); however, much of the variation was explained by differences across organizations. The organizational factors that contribute to such variation in information technology use are not well understood. This gap in knowledge provided the impetus for the current study.

The purpose of this study was to investigate the role of organizational context and nurse characteristics in explaining variation in nurses’ use of PDAs and mobile Tablet PCs for accessing evidence-based information. The goal was to identify success factors that supported diffusion of technology and evidence-based practice.

### Theoretical perspective

The Promoting Action on Research Implementation in Health Services (PARIHS) model [7,8] provided the framework for studying the impact of providing nurses with PDA-supported, evidence-based practice resources, and for studying the organizational, technological, and human resource variables that impact nurses’ use patterns. The successful implementation of evidence into practice is conceptualized to be a function of the relationship between: the nature of the evidence; the context in which practice change will occur (prevailing culture, the leadership roles assigned, and measurement and feedback); and the mechanisms by which the change is facilitated [7,8]. The PARIHS model was used in a multi-level modeling study of the nursing and institutional factors that explain variation in research utilization at the nurse, specialty, and hospital levels [9]. The largest proportion of variation in research utilization was explained by individual level variables. However, both specialty and hospital level variables contributed a small but significant proportion of the variance in research utilization, indicating that organization context factors matter to some extent [9]. The following variables explained variation in research utilization at the specialty level: relational capital, facilitation, nurse-to-nurse collaboration, and autonomy. Furthermore, in contexts where nurses perceived more favourable culture, leadership, and evaluation, research use was, on average, higher than among those with low perceptions of their context. At the hospital level, innovative organization, responsive administration, and staffing support were significant predictors of research utilization [9]. In another multi-level analysis of variables derived from the PARIHS model by the same group of researchers, hospital characteristics that positively influenced nurses’ research utilization were staff development, opportunity for nurse-to-nurse collaboration, and staffing and support services [10]. Increased emotional exhaustion was associated with less reported research use. Nurses working in contexts with more positive culture, leadership, and evaluation reported significantly more research utilization. The PARIHS model and this previous research guided the research questions in the present study.

### Research Questions

This study was designed to answer the following research questions:

1. What are the frequencies of use of the three different types of evidence; namely, RNAO BPGs, Nursing Pus, and Lexi/PEPID resources?

2. How does organizational context—specifically, presence of electronic documentation, leadership, culture, opportunity for evaluation feedback, formal interactions, informal interactions, structural and electronic resources, and organizational slack—explain variation in the frequency with which nurses use PDAs (or Tablet PCs) to access information resources?

3. How do nurse characteristics—specifically experience in the current unit, education, practice role, attitude towards research, belief about research use, problem solving style, and burnout—explain variation in the frequency with which nurses use PDAs (or Tablet PCs) to access information resources?

### Related literature

Recognition of the value of PDAs as an information and communication tool in nursing practice is relatively recent [11-16]. Research by Hardwick *et al*. (2007) suggests that handheld technology can be used by nurses to improve and streamline patient care by capturing clinical data, by organizing and reporting home health services, and by providing references for evidence-based practice [17]. Doran (2009) found that PDAs can be useful for promoting timely communication, enabling evidence-based collaborative practice, and for supporting workplace learning [15].

Stolee *et al*. (2010) reviewed the literature on the use of electronic health information systems in home care. Training requirements was one of the most prevalent barriers identified. Of note, the top three facilitators for the use of information systems in home care were portable technology, strategies for decreasing data entry errors, and managerial support during the initial phases of implementation [18]. These facilitators and barriers illustrate the need for a coordinated effort when implementing new technologies and information systems, and employment of user-friendly, portable modes of information access. Information use was associated with perceived benefits of using information systems and satisfaction with the system. Another literature review, also published in 2010, concluded that computer experience, system design, and system performance, such as system speed, were the primary factors that influenced nurses’ attitudes toward healthcare information technology [19]. A survey of 201 registered nurses (RNs) in the state of Ohio found computer experience, user involvement, and nursing management support significantly explained information system use [20].

Research in the United Kingdom by Honeybourne *et al.* (2006) examined the impact of PDAs on patient care by identifying how often clinical staff accessed the materials available to them to inform clinical decision making. The research sample included nursing staff and participants from other health disciplines. The authors concluded that all participants used PDAs but in varying frequencies. Eleven of the twelve staff reported a benefit of handheld systems in addressing immediate patient concerns. Eleven of the twelve staff reported that the PDA was useful in supporting their educational needs. They identified that a key point in providing evidence at the point of care is the speed at which the information can be delivered. The resources most frequently referenced in the clinical setting were: drug reference information, medical calculators, guidelines information, and administrative tasks [11]. While the Honeybourne *et al*. study was useful for clarifying benefits of using PDAs in clinical practice, it was limited by small sample size. A Canadian study addressed this limitation by including a sample of 488 nurses from 29 acute care, home care, LTC, and correctional settings [6]. The authors reported a significant reduction in perceived barriers to research utilization following the introduction of PDAs for nurses working in acute care, home care, and LTC settings [6]. As nurses used the PDA or Tablet PC devices, they felt a significant improvement in their own research values, skills, and awareness, as well in the presentation and accessibility to research evidence. There was a significant improvement in perceptions of the quality of care and job satisfaction for PDA users, but not for Tablet PC users, suggesting outcomes varied by device type [6]. The current study was designed to advance this previous research by investigating the role of organizational context and nurse characteristics in explaining variation in nurses’ use of PDAs and mobile Tablet PCs for accessing evidence-based information.

## Methods

### Study design

A survey design was used, involving baseline (T1) questionnaire and follow-up (T2) questionnaire at approximately six months post-implementation, to answer the research questions.

### Setting and sample

The setting included 24 organizations representing three sectors: hospitals, LTC facilities, and community organizations (home care and public health). Each organization independently determined which nurses would be provided with access to the mobile devices and which devices would be used. All of the nurses who were using PDAs or Tablet PCs as part of the Ministry of Health and Long-Term Care Phase 2 PDA Initiative were eligible to participate in the study. The total sample consisted of 710 participants (response rate 58%) at Time 1 and 469 participants at Time 2 (response rate 66%). The study received ethics approval from the research ethics office at the University of Toronto and from each participating organization.

### Implementation

Organizations participating in the Ministry of Health and Long-Term care Nursing Secretariat’s Phase 2 PDA Initiative provided their nurses with a personal PDA or a shared Tablet PC that enabled access to electronic information resources. Organizations were responsible for configuring the PDAs/Tablet PCs to enable access to the various resources either using the healthcare organization’s local area network or through a mobile cellular network. Some but not all organizations provided nurses with corporate e-mail accounts, a new service for many nurses, and a pre-requisite for using all of the features of Nursing Plus, which sends e-mail alerts to registered users with links to journal abstracts on topics selected by the nurse. Education and facilitation were provided by the RNAO through a ‘train-the-trainer model’ to prepare organizational facilitators, webinars, and electronic technical support. Each site then developed a strategy for teaching and supporting its nurses.

### Procedure for data collection

#### Recruitment process

Each participating organization provided a list of the nurses who were expected to participate in the PDA initiative. An information package prepared by the research team was distributed to eligible nurses by the site liaisons. The package included a baseline questionnaire and two copies of the information letter/consent. It also included two self-addressed stamped envelopes, one to return the questionnaire and a second to return the consent form. Approximately three weeks after the packages were distributed, a thank you/reminder was distributed, and duplicate packages were sent to non-respondents approximately three weeks later.

#### Data collection tools

Data from the T1 questionnaire were linked with the T2 data for each participant. Data collection occurred from June 2009 to March 2010. The questionnaires and Cronbach’s alpha for each subscale measuring the dependent and independent variables are summarized in Table 1 (electronically accessible as an additional file). All of the survey tools have been used in previous research describe below, with the exception of breadth of device use, and thus were not pilot tested prior to use in this study. The dependent variables in the study were frequency of use of information technologies (*i.e.*, PDAs or Tablet PCs) and frequency of use of the three different forms of evidence (*i.e.*, RNAO BPGs, Nursing Plus, and Lexi/PEPID reference resources). Nurses were asked to respond to a series of questions on the T2 questionnaire to assess the frequency with which they used the PDA/Tablet PC and information resources.

**Table 1:** Variables and questionnaires used in the study

**Concept**	**Sample item**	**Response format**	**Cronbach’s Alpha**
**Dependent Variables**			
Frequency of PDA/ Tablet PC Use	“How often did you use a PDA or Tablet PC as part of the PDA Initiative?”	“0=never”,“1=almost never”,“2=every few days”,“3=daily”,“4=more than once a day”	Single item
Frequency of use of RNAO BPGs	“For each information resource, please indicate how frequently you accessed it during a typical week.”	“0=never”,“1=almost never”,“2=every few days”,“3=daily”,“4=more than once a day”	Single item
Frequency of use of Nursing PLUS	“For each information resource, please indicate how frequently you accessed it during a typical week.”	“0=never”,“1=almost never”,“2=every few days”,“3=daily”,“4=more than once a day”	Single item
Frequency of use of Lexi or PEPID drug and medical reference guide	“For each information resource, please indicate how frequently you accessed it during a typical week.”	“0=never”,“1=almost never”,“2=every few days”,“3=daily”,“4=more than once a day”	Single item
**Independent Variables**			
**Organizational Context**			
Alberta Context Tool (Estabrooks, 2009)			
Leadership (6 items)	“Focuses on successes rather than failures.”	“1=strongly disagree”, “2=disagree”,“3=neither agree or disagree”, “4=agree”,“5=strong agree”.	0.91
Culture (6 items)	“I receive recognition from others about my work.”	“1=strongly disagree”, “2=disagree”,“3=neither agree or disagree”, “4=agree”,“5=strong agree”.	0.79
Feedback processes/evaluation (6 items)	“Our team routinely monitors our performance with respect to the action plans.”	“1=strongly disagree”,“2=disagree”,“3=neither agree or disagree”, “4=agree”,“5=strong agree”.	0.92
Informal interaction (9 items)	“How often did you have a patient care related discussion with other nurses (RNs or RPNs).”	“never=0 time”,“rarely=1-5 time”,“occasionally=6-10 time”,“frequently=11-15 times”,“almost always=16 time or more”.	NA
Formal interaction (4 items)	“In the last typical month, how often did you participate in team meetings.”	“never=0 time”,“rarely=1-5 time”,“occasionally=6-10 time”,“frequently=11-15 times”,“almost always=16 time or more”.	NA
Social capital (6 items)	“People in the group share information with others in the group.”	“1=strongly disagree”,“2=disagree”,“3=neither agree or disagree”,“4=agree”,“5=strong agree”.	0.8
Structural and electronic resources (11 items)	“In the last typical month, how often did you use a library?”	“never=0 time”,“rarely=1-5 time”,“occasionally=6-10 time”,“frequently=11-15 times”,“almost always=16 time or more”.	NA
Slack, staffing (3 items)	“On my unit we have enough staff to get the *necessary w*ork done.”	“1=strongly disagree”,“2=disagree”,“3=neither agree or disagree”, “4=agree”,“5=strong agree”.	0.88
Slack, space (3 items)	“On my unit we have adequate *space* to provide patient care.”	“1=strongly disagree”, “2=disagree”, “3=neither agree or disagree”, “4=agree”, “5=strong agree”.	0.76
Slack, Time	“How often do you have time to do something extra for patients?”	“1=never”, “2=rarely”,“3=occasionally”,“4=frequently”,“5=almost always”.	0.8
Organizational support (Estabrooks, 1997)	“There is support for innovative ideas about patient care.”	“1=strongly disagree”,“2=disagree”,“3=neither agree or disagree”,“4=agree”,“5=strong agree”.	Single item
Breadth device functions	“Did you use the PDA or Tablet for recording vital signs?”	0=No;1=Yes	NA
**Individual Nurse Characteristic Variables**			
Attitude toward research (Lacey, 1994)	“Research is needed to continually improve clinical practice.”	“1=strongly disagree”,“2=disagree”,“3=neither agree or disagree”,“4=agree”,“5=strong agree”.	0.81
Belief willing to implement research (Estabrooks, 1997)	“I am willing to implement research when it contradicts something I learned in nursing school.”	“1=strongly disagree”,“2=disagree”,“3=neither agree or disagree”,“4=agree”,“5=strong agree”.	0.8
Belief, actually implements research (Estabrooks, 1997)	“How often do you actually implement research when it contradicts something you learned in nursing school.”	“never=0 time”,“rarely=1-5 time”,“occasionally=6-10 time”,“frequently=11-15 times”,“almost always=16 time or more”.	0.87
Problem-solving (Heppner, 1986)	“When I have a problem, I think of as many possible ways to handle it as I can until I can’t come up with any more ideas.”	“1=strongly disagree”,“2=disagree”,“3=neither agree or disagree”,“4=agree”,“5=strong agree”.	0.72
Emotional exhaustion (3 items) Maslach Burnout Inventory(Schaufeli, et al., 1986)	“I feel burned out from my work.”	“0=never”,“1=a few times a year or less”,“2=once a month or less”,“3=a few times a month”, “4=once a week”,“5=a few times a week”, “6=every day”.	0.86
Cynicism (3 items) (Schaufeli, et al., 1986)	“I have become more cynical about whether my work contributes to anything.”	“0=never”,“1=a few times a year or less”,“2=once a month or less”,“3=a few times a month”, “4=once a week”,“5=a few times a week”, “6=every day”.	0.75
Professional efficacy (3 items) (Schaufeli, et al., 1986)	“I feel exhilarated when I accomplish something at work.”	“0=never”,“1=a few times a year or less”,“2=once a month or less”,“3=a few times a month”, “4=once a week”,“5=a few times a week”, “6=every day”.	0.76

The PARIHS model guided the selection of independent variables for measurement in this study. More specifically, successful implementation of evidence into practice is conceptualized to be a function of the context in which practice change will occur (prevailing culture, the leadership roles assigned, and measurement and feedback) [7,8]. The context variables were measured with the Alberta Context Tool (ACT) [21], described below. In addition, we measured nurses perception of organizational support [22]; attitude towards research [23]; belief, willingness to implement research [22]; belief, actually implement research [22]; problem-solving style [24]; and burnout measured with the short version of the Maslach Burnout Inventory [25,26]. These individual characteristics have been found to influence variation in research use in previous research [9,27,28]. For example, critical thinking dispositions, attitudes toward research use, belief suspension, and intent to use research differentiated inpatient units with high research utilization from those with low research utilization [23]. A variable labeled ‘breadth of device functions’ was included, reasoning that higher use would be expected where the PDA or Tablet PC device was used for more patient care functions. Breadth of device functions was created as an ordinal variable to represent the different device functions available to nurses within their practice settings. With the exception of the information resources that were available to nurses in all organizations, the other types of functions available to nurses on their devices were at the discretion of organizations. Other device functions available to nurses ranged from access to email, clinical documentation, and laboratory and radiology results. A variable was created to represent the breadth of functions available by counting the number of functions nurses reported on the follow-up survey.

#### Organizational context

The ACT, a 58-item instrument was developed by Estabrooks *et al.*[21] to measure organizational context. The 58 items include variables of: leadership (six items), culture (six items), feedback processes/evaluation (six items), organizational resources (11 items); organizational slack including time (four items), space (three items) and staffing (three items); informal interactions (nine items), formal interactions (four items), and social capital (six items). Cronbach’s alpha have been reported as ranging from 0.54 to 0.91 for a 13-concept version [21] and from 0.37 to 0.92 for a 10-concept version [29]. Construct validity was established with confirmatory factor analysis [29]. In addition eight of the ten ACT concepts were statistically significantly associated with research utilization among healthcare aides working in LTC facilities. The majority of the ACT concepts also showed a statistically significant trend of increasing mean scores when arrayed across the lowest to the highest levels of instrumental research use [29]. Significant between unit variation on each of the 10 ACT concepts was reported for Canadian acute paediatric settings, even after controlling for individual characteristics [30].

#### Burnout

The short version of the Maslach Burnout Inventory – General Survey (MBI-GS) [25,26] was used to measure burnout. It consists of nine items that measure three aspects of burnout, emotional exhaustion (three items), depersonalization (three items), and decreased personal accomplishments (three items), with less skewed distribution than the original MBI Human Service Survey [31]. A higher score on emotional exhaustion and cynicism, and a lower score on professional efficacy, indicate a high level of burnout. Psychometric properties of the MBI-GS are well established, and burnout measured with the nine-item version was associated with physician workload and value congruence [31]. The Cronbach’s alpha in this study are reported in Table 1.

#### Perception of problem-solving ability

Heppner (1986) developed the Problem Solving Inventory (PSI) to measure an individuals’ perception of their problem-solving skills. The original scale consisted of three dimensions of perception of problem-solving ability—problem-solving confidence, avoidance style, and personal control—with good internal consistency across a number of populations and cultures [32-35] with average alpha coefficient in the high 0.80s. A modified 10-item version was used in this study. Example item and Cronbach’s alpha are presented in Table 1. Concurrent, discriminate, and construct validity have been assessed across various research studies [32-35].

#### Data analysis

Analysis was conducted on the 469 participants for whom Time 1 and Time 2 data were available. The demographic characteristics of participants were summarized using descriptive analysis. The results were expressed as mean and standard deviation (SD) for continuous variables and count (percentage) for categorical variables. Chi-squared tests were applied for comparison of differences in device use. The survey measurements were conducted on individual nurses nested within organizations that were in turn nested within healthcare sectors. The Intraclass Correlation (ICC) was introduced to assess the similarity of individual nurses within cluster at each level, and design effect (DE) was computed based on the average size of clusters at each level. The hierarchical random-effect regression approaches were constructed to explain this multilevel data structure. The dependent variables used in the regression analysis were defined in Table 1.

All statistical tests were two-sided at the 0.05 significant level. The results from all regression models were expressed as estimates and corresponding two-sided 95% confidence intervals (CIs) along with associated p-values. All estimates and CIs were reported to two decimal places. The goodness of fit was assessed using the ratio of the generalized chi-square statistics to its degrees of freedom and Q-Q plot. The Chi-squared tests and t-tests were conducted using IBM SPSS v19 and all regression models were performed using SAS 9.3.

## Results

### Demographic characteristics

Of the 469 participants from whom complete T1 and T2 data were available, there were 350 (74.6%) RNs, 15 (3.2%) nurse practitioners, and 99 (21.1%) registered practical nurses and five (1.1%) participants who did not report their professional designations. The ‘average’ participant was a 44.7 (SD 10.2) year-old female with 14.9 (11.4) years of experience in nursing, 7.6 (7.0) years on current unit and 11.3 (9.7) years in current organization. The majority of nurses (76.0%) worked full-time, an average of 37.7 (9.6) hours each week. The majority of participants (59.1%) had received a diploma as their highest level of nursing education. There were 29.9% with a baccalaureate degree and 9.4% with a masters or doctorate degree.

### Devices used

Device use was assessed at time two survey. Handheld devices (PDA, Smartphone, BlackBerry *et al*.) were reportedly used by 152 participants and Tablet PCs (including Netbooks and laptops) were used by 165 participants. A small group of 36 participants used both a PDA and a Tablet PC device as part of the PDA initiative. Handheld PDAs were more frequently used in hospitals, and Tablet PCs were more common in LTC and community settings.

### Results: Research Questions

#### Research question one

The first research question asked, **‘**What are the frequencies of use of the three nursing electronic resources?’ The results are summarized in Table 2 and 3. Almost 60% of the nurses used a Tablet PC device at least once very few days or more often, and 72% used a PDA every few days or more often. The Chi square result indicates PDA users reported using their device more frequently than Tablet PC users. Eighty-nine nurses reported reasons why they ‘never’ used a PDA or Tablet PC during the study. Such reasons included equipment not working properly, takes too much time, difficult to access, lack of training, lack of perceived need, and device not available.

**Table 2:** Use of PDA/Tablet PC devices during study

	**Tablet PC device users**		**PDA device users**	
**Used a mobile device**^**a**^	**n**	**%**	**n**	**%**
Several times a day	32	19.51	55	36.67
About once a day	21	12.81	21	14.00
Once every few days	49	29.88	32	21.33
Almost never	62	37.80	42	28.00
Never^**^	0	0.00	0	0.00
Total number of valid responses	164	100.00	150	100.00

Chi-square: P=0.001^b^.

^a^There were 119 respondents who failed to report device use and 36 respondents indicated they used both a PDA and Tablet PC. **97 respondents indicated they never used a mobile device during the study. So we did not count them as the participants of this study.

^b^For these 97 respondents, their responses were not included in the Chi-square analysis because it was not possible to determine which type of device they had been provided.

**Table 3:** Frequency of nurses’ use of information resources

**Resource**	**Tablet PC**	**PDA device**	***p *value **^**b**^
	**N **^**a **^**(%)**	**N (%)**	
Google	82 (61.65)	51 (38.35)	0.0072
IV compatibility	17 (37.78)	28 (62.22)	0.1011
Medical diagnoses, reference information	58 (45.67)	69 (54.33)	0.3290
Drug dictionary	48 (40.68)	70 (59.33)	0.0428
In-house resources	60 (62.50)	36 (37.50)	0.0143
Nursing+ email alerts	58 (59.79)	39 (40.21)	0.0537
RNAO best practice guidelines	51 (63.13)	45 (46.88)	0.5403
Laboratory values	39 (45.88)	46 (54.12)	0.4477
Calculator	29 (40.28)	43 (59.72)	0.0990
Nursing+ database search	40 (57.97)	29 (42.03)	0.1854

^a^ N’s vary depending on the number of nurses indicating they used the information resource every few days or more often.

^b^*p* value from Chi-square test.

#### Information Resources

Nurses were provided with three core information resources: a drug and medical reference book (PEPID or Lexi), RNAO Best Practice Guidelines, and Nursing Plus. Most organizations chose to add additional resources such as corporate policies, clinical documentation, and other in-house resources. Nurses were asked to record how often they used their PDA/Tablet PC to access each resource during a typical week. Table 3 shows the percentage of nurses who accessed each resource at least once every few days or more often. It shows that nurses accessed Google most frequently, followed closely by medical and drug reference information.

The results in Table 3 indicate that Tablet PC type devices were used more frequently to access Google, and in-house resources. No differences were observed between Tablet PC and PDA users to access RNAO best practice guidelines. PDA users were more likely to use their device to access the drug reference than Tablet PC users.

#### Research question two and three

The second research question asked, **‘**how does organizational context features explain variation in the frequency with which nurses use PDAs (or Tablet PCs) to access information resources?’ The third research question asked, ‘how do individual characteristics explain variation in the frequency with which nurses use PDAs or Tablet PCs to access information resources?’ To address questions two and three, the ICCs and DEs for outcome variable at each level were computed and are shown in Table 4. At the organization level, the ICCs for the four outcome variables were greater than 0.10 [36], and both DEs calculated based on the average sample size of 29 were larger than 2 [37]. At sector level, the ICC for predictor of ‘How often use a PDA or Tablet PC’ was larger than 0.1. Although the ICC for RNAO BPG was close to 0, it was 0.11 at organization level. Therefore, a hierarchical regression model (HLM) was necessary to account for the multilevel nature of the data.

**Table 4:** **ICCs and DEs for outcome variables at each level**^**a**^

**Level**	**Variable**	**ICC (95% CI)**	**DE**
Organization level	How often use a PDA or Table PC	0.35 (0.22, 0.50)	10.63
	RNAO best practice guideline use	0.11 (0.05, 0.23)	4.03
	Best evidence Nursing+ database use	0.11 (0.05, 0.23)	4.08
	Drug dictionary (PEPID/Lexi)	0.31 (0.18, 0.46)	9.57
Sector level	How often use a PDA or Table PC	0.13 (0.03, 0.45)	32.98
	RNAO best practice guideline use	0	1
	Best evidence Nursing+ database use	0	1
	Drug dictionary (PEPID/Lexi)	0.01 (0.00, 0.16)	3.46

^a^ICCs were used to determine if substantial variation exists between groups compared to variation within groups.

The results from the three-level random-effect model indicate that two predictors had a significant effect on frequency of device use (see Table 5 for details). For those nurses having high scores on ‘Breadth of Device Functions,’ frequency of device use was also high. Nurses using a PDA had higher frequency of use than those using a Tablet PC. The analysis of use of RNAO BPG revealed that six predictors were significantly associated with the frequency of BPG use. Nurses who were willing to implement research were likely to use BPGs. Those who reported a high score on ‘ACT Structural and Electronic Resources,’ ‘ACT organizational slack time’ or ‘Breadth of Device Functions’ also had a high frequency of BPG use. However, for ‘ACT organizational slack staff’ a high score was associated with a low frequency of BPG use.

**Table 5:** Estimates and corresponding 95% confidence intervals (CIs) for outcomes

**Outcome**	**Predictor**	**Estimate (95% CI)**	***p *value**
Frequency of device use	Device	0.36 (0.05, 0.68)	0.0246
	Breadth device functions	0.24 (0.17, 0.31)	<0.0001
Frequency of BPG use	Belief willing to implement research	0.17 (0.03, 0.31)	0.0157
	ACT Structural and electronic resources	0.17 (0.08, 0.26)	0.0007
	ACT Organizational slack staff	−0.12 (−0.21, -0.02)	0.0178
	ACT Organizational slack time	0.17 (0.02, 0.33)	0.0280
	Breadth device functions	0.08 (0.03, 0.13)	0.0039
Frequency of Use for Nursing+ Searchable Database of Journal Abstracts	ACT Culture	0.17 (0.01, 0.33)	0.0431
	ACT Structural and electronic resources	0.14 (0.05, 0.23)	0.0049
	Breadth device functions	0.14 (0.09, 0.19)	<0.0001
Frequency of Lexi/PEPID Drug Dictionary Use	ACT Culture	0.30 (0.11, 0.48)	0.0030
	ACT Organizational slack staff	−0.14 (−0.26, -0.03)	0.0141
	Breadth device functions	0.09 (0.03, 0.15)	0.0020

The analysis of use of Best Evidence for Nursing Plus Database revealed that three predictors were significantly associated with the frequency of Nursing Plus database (see Table 5 for details). Nurses who reported a high score on ‘ACT Structural and Electronic Resources,’ ‘ACT Culture’ and ‘Breadth of Device Functions’ also had a high frequency of Nursing Plus database use.

The analysis of use of Lexi/PEPID resources revealed three factors were significantly associated with frequency of drug dictionary use (see Table 5 for details). ‘ACT organizational culture’ and ‘Breadth of Device Functions’ were associated with high frequency of Lexi/PEPID drug dictionary use. ‘ACT organizational slack staff’ was associated with low frequency of use.

**Figure 1:**
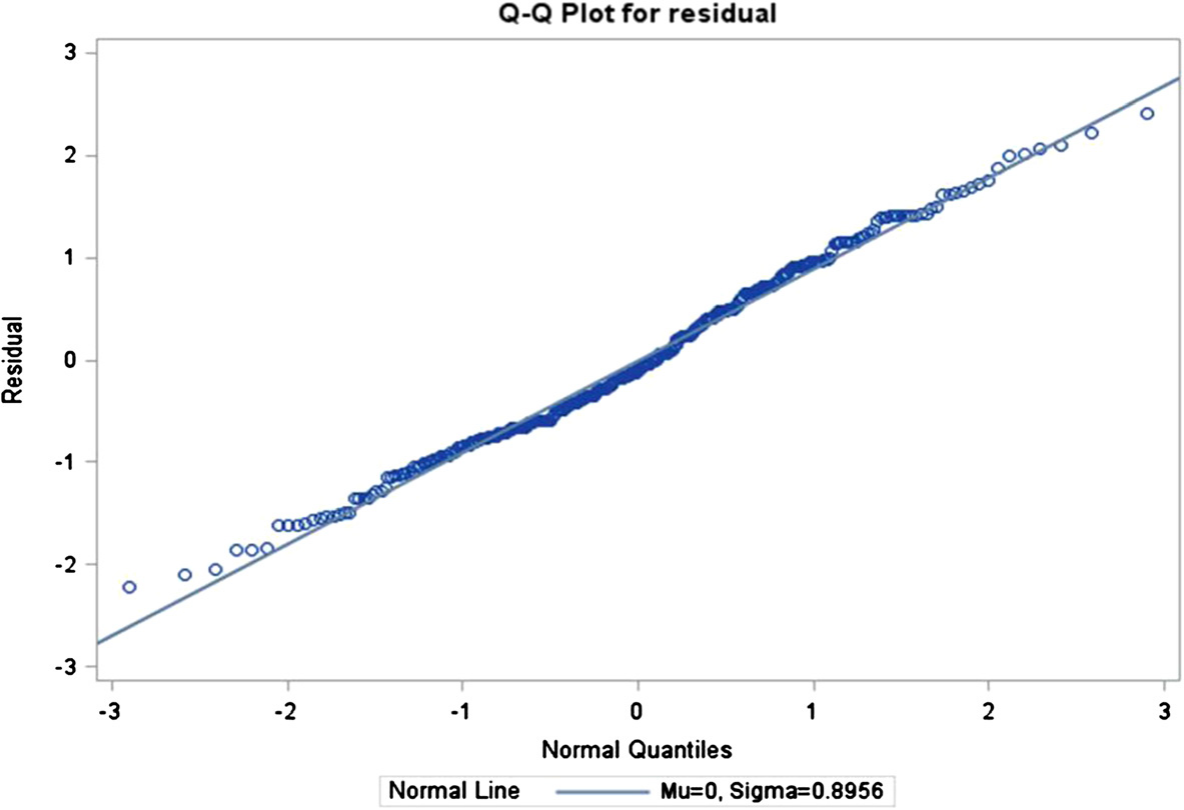
Q-Q plot for the model of frequency of PDA use.

**Figure 2:**
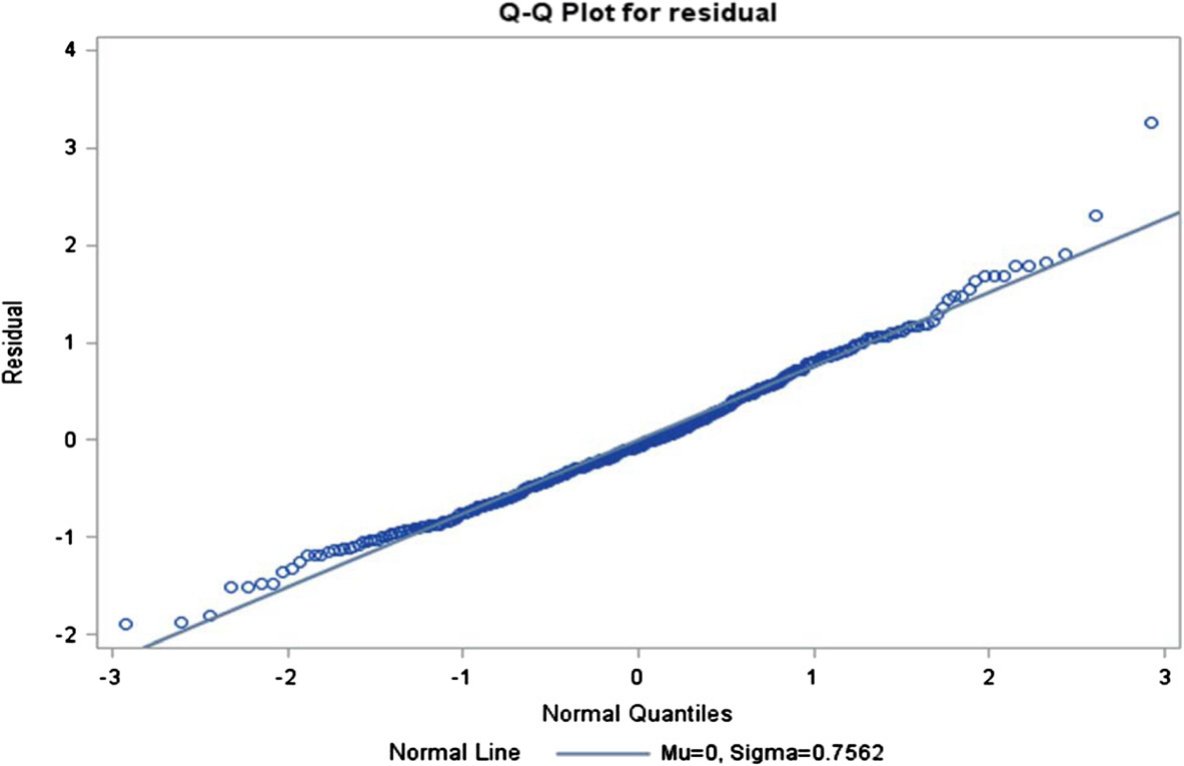
Q-Q plot for the model of frequency of best practice guideline use.

**Figure 3:**
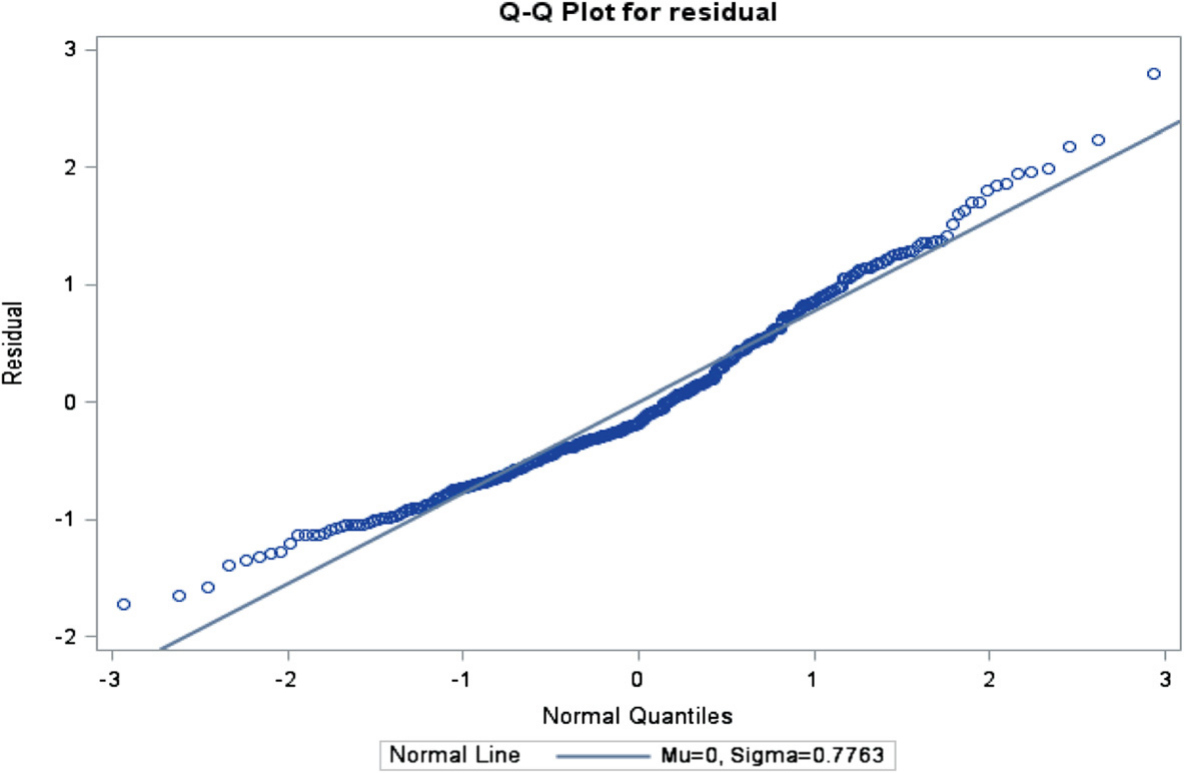
Q-Q plot for the model of frequency of use of best evidence for nursing+ searchable database of journal abstracts.

**Figure 4:**
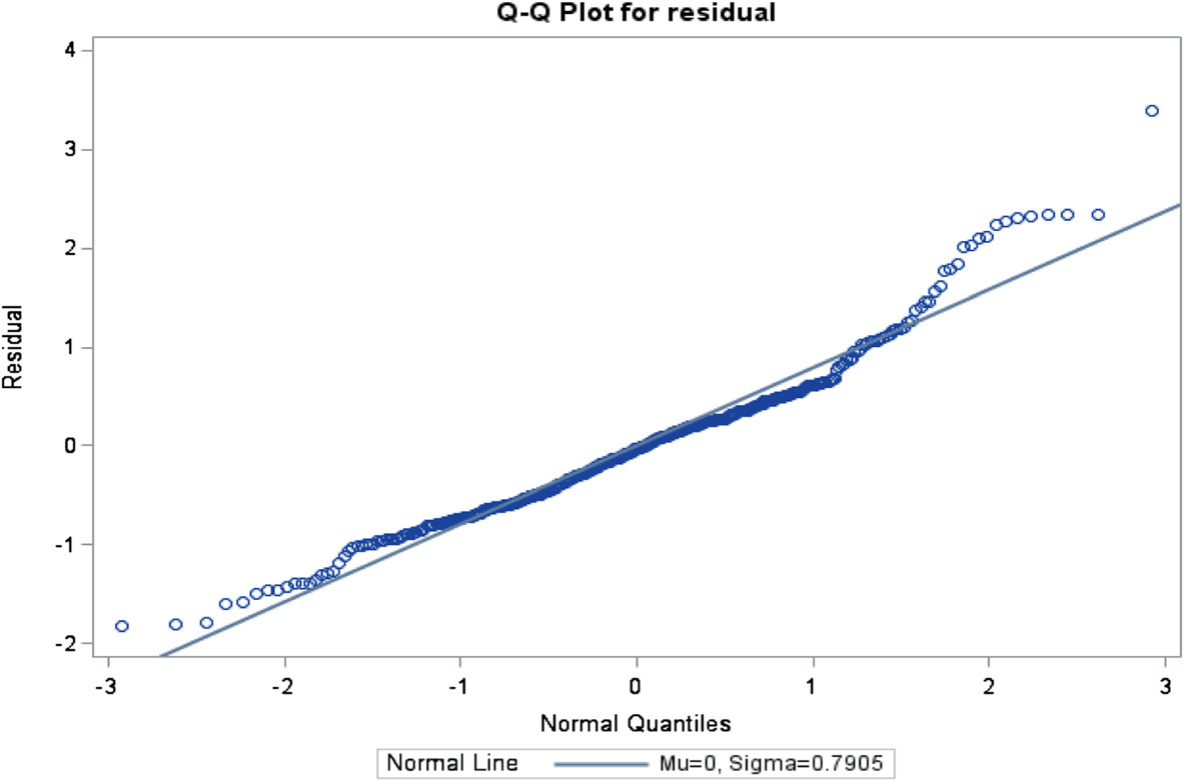
Q-Q plot for the model of frequency of use of Lexi/PEPID drug dictionary.

The ratios of generalized chi-square statistics to their degrees of freedom were 0.85, 0.61, 0.63 and 0.67 for these four models respectively, which were close to 1 [38], indicating that the variability in these data has been properly modeled, and that there was no residual over dispersion. The Q-Q plots in Figures 1, 2, 3 and 4 for these models showed that all points were normally distributed, indicating a good fit for the data.

## Discussion

### Study limitations

Organizations chose to participate in the Ministry of Health and Long-Term Care PDA initiative, and were at liberty to select the device type they would provide nurses. Several institutions had implementation problems and poor technical support for device-specific activation, training, internet access, and allowing/providing staff with individual e-mail addresses. There was no control group and therefore it was not possible to rule out other possible variables that could explain change in information use. Only 58% of nurses responded to the baseline (Time 1) survey and only 66% of these responded to the follow-up (Time 2) survey. Comparisons between both respondents at Time 1 and Time 2 and the full target samples—College of Nurses of Ontario (CNO) registration database for 2009—indicated the respondent groups to be younger, to have less years of experience in nursing, and to have more highly educated nurses (see Table 6). A response bias could have impacted the study findings. Effects on knowledge, practice, and health outcomes were not assessed in this study.

**Table 6:** **Comparisons of demographic characteristics of respondents at both Time 1 and time 2 and CNO database**^**a**^

**Variable**	**Mean (SD)/Count (Proportion)**			***p *value**		
	**T1**^**b**^	**T2**^**b**^	**CNO**	**T1 vs CNO**	**T2 vs CNO**	**T1 vs T2**
Age	43.4 (10.8)	44.7 (10.2)	45.88 (12.0)	<0.0001	0.0487	0.0425
Experience in nursing	13.3 (10.8)	14.9 (11.4)	18.61 (12.9)	<0.0001	0.0009	0.1887
Female	680 (95.9)	443 (95.9)	144167 (94.6)	0.8524	0.8822	0.9850
Professional designationRN	527 (73.7)	350 (75.4)	114625 (75.7)	<0.0001	<0.0001	0.3239
RPN	168 (23.5)	99 (21.3)	36574 (24.0)			
RN (EC)	20 (2.8)	15 (3.2)	1206 (0.8)			
EducationDiploma/Certificate	422 (59.3)	277 (60.1)	97857 (68.7)	<0.0001	<0.0001	0.6458
Bachelors	229 (32.2)	140 (30.4)	36745 (25.8)			
Masters and Doctorate	61 (8.6)	44 (9.6)	7841 (5.5)			

T1=Time 1, T2=Time 2, and CNO=College of Nurse of Ontario database.

^a^t test for age and Experience in nursing, binomial proportion test for Female, and chi-square test for Education.

^b^Calculations were based on the number of surveys returned with valid response at Time 1 and Time 2.

### Discussion of research findings

The study investigated how organizational context and individual characteristics explain variation in use of PDAs (and Tablet PCs) to access information resources in clinical practice. The research findings suggest that mobile technologies have the potential to impact research utilization.

### Frequency of use

Prior to the study, 73% of the nurses had not used a PDA for personal or work use, and 78% had not used a Tablet PC. At the conclusion of the study, 53% of the nurse respondents indicated they were using a PDA or Tablet PC once every few days or more often, and 20% indicated that they never used it. Thus, the majority of nurses reported a significant increase in use of PDA or Tablet PCs following their participation in the Ministry of Health and Long-Term Care PDA initiative. Those nurses who indicated they never used the device reported issues such as equipment not working properly, takes too much time, difficult to access, lack of training, lack of perceived need, and device not available. As noted above, some institutions had implementation problems, including lack of training and technical support, which could explain why PDAs and Tablet PC devices were not used or under used by some nurses. It is important that attention be paid to organizational readiness for technology innovation before implementing mobile technologies designed to support nurses’ information use.

The frequency with which nurses used their PDAs or Tablet PCs was associated with the breadth of functions available on the device and the device type. Nurses who had PDAs reported using their device more often than nurses who had Tablet PCs. This could be explained by the observation that each nurse was assigned her own PDA whereas, those with Tablet PCs often shared the device with other nurses, which could have resulted in less accessibility when needed. Similar findings were reported by Doran *et al*. (2010) in an earlier study who also reported significant improvements in barriers to research utilization, quality of care, and job satisfaction for PDA users but not for Tablet PC users.

### Information resources

There have been a number of studies that have explored the various resources that nurses utilize and the kinds of knowledge they require in their day-to-day work [39-41]. Royle *et al*. found that, to access professional information, two-thirds of the nurses in their study consulted with colleagues daily, most used reference sources and textbooks weekly, and two-thirds of them read journal articles monthly [39]. Thompson *et al.* found that nurses preferred human sources of information and that colleagues, other members of the primary care team, or senior members of the clinical team were viewed as the most useful and accessible information sources [41]. In another study, observation of nurses’ information-seeking behaviour through work-sampling methodology confirmed that nurses most often sought information from colleagues [14]. Nurses expressed stronger preferences for reference information and procedural information than for research information. Hospital nurses’ top priorities for information resources at the point-of-care were information on intravenous (IV) drug compatibility, a drug dictionary, and IV medication protocols. Access to the types of information identified by these nurses is now readily available on PDAs and other mobile technologies, such as Tablet PCs [42]. In the current study, nurses indicated that they accessed Google most often for information, followed closely by medical and drug reference information. These findings are remarkably similar to the findings reported by Doran *et al*. (2010) in an earlier study involving a different set of organizations and different group of nurses. In that previous study, the most frequently used information resources were drug reference information, medical reference information, and Google search engine. In the current study a higher proportion of nurses reported using the RNAO BPG every few days or more often than what was observed in the previous research [6] (31% compared to 25%, respectively). This could reflect a trend toward increased use of RNAO guidelines as more organizations in Ontario have promoted BPG use.

### Organizational context

Rycroft-Malone defined organizational context as ‘the environment or setting in which people receive healthcare services’ [43] (p.229). The organizational context is widely considered to be an important influence on the implementation of research evidence in healthcare settings [44]. Estabrooks *et al*. developed the ACT to measure ten dimensions of the organizational context: culture, leadership, evaluation, social capital, informal interactions, formal interactions, structural and electronic resources, and organizational slack time, staff, and space [21]. The dimensions were found to be associated with instrumental research use [44]. In this present study, four dimensions of the ACT were associated with frequency of information resource use; specifically, structural and electronic resources, organizational culture, organizational slack time, and organizational slack staff. Slack staff was negatively associated with increased BPG use, whereas structural and electronic resources and slack time were positively associated with increased BPG use. Access to structural and electronic resources and organizational culture were positively associated with increased use of Best Evidence for Nursing Plus database. Organizational culture and slack staff resources were associated with Lexi/PEPID drug dictionary use. These features of the organizational context are modifiable and could therefore be the focus of organizational interventions to increase research utilization. Similar findings were reported by Randell and Dowding who also noted a key element for the successful introduction of clinical decision support technology was clinician engagement [45]. They noted that successful implementation of clinical decision support technology was associated with the need for adequate resources, characteristics of the system, a supportive environment, and adequate training; key features also found important in our study.

The negative relationship between slack staff and RNAO BPG use and Lexi/PEPID use was counter-intuitive, but could be explained by the fact that in organizations where nurses had access to excess staff they relied more on staff for their information needs than in organizations where nurses had access to fewer staff, resulting in greater use of the PDA (or Tablet PC) to meet information needs. Interestingly, the breadth of device functions to which the nurse had access, such as clinical documentation, email, and phone, was associated with high use of RNAO BPGs, Nursing Plus database, and Lexi/PEPID drug dictionary. It is possible that as technology becomes fully embedded into the activities of the organization, there is greater propensity to use the technology for information seeking than where it is less embedded.

### Individual nurse determinants of research use

One individual nurse characteristic variable was strongly associated with RNAO BPG use in the HLM analysis; specifically willingness to implement research. These findings underscore the complexity of variables that operate at organizational and individual levels to explain variation in evidence-based practice. Future research is needed, involving a larger number of healthcare organizations and sample of nurses, to more fully explore how organizational factors and individual characteristics interact to influence the use of information technologies to support evidence-based practice.

### Study implications

1. Handheld portable devices support nurses’ use of information resources in clinical practice.

2. Nurses should be provided with access to electronic resources such as drug and medical reference information, and to best practice information to support their learning needs and to promote evidence-based practice.

3. Technology innovation designed to support evidence-based practice should adopt change strategies aimed at enhancing organizational culture, improving access to structural and electronic resources, and ensuring nurses have time to access information resources at the point-of-care.

4. Future research should investigate the interaction between organizational context variables and individual factors. Where nurses share technology such as Tablet PCs, it would be important to assess the degree to which nurses have access to the technology and the barriers to its use.

## Conclusions

Use of PDAs and Tablet PCs for accessing information resources supported nurses’ self-reported use of information resources. Several of the organizational context variables explained variation in the frequency of PDA/Tablet PC use to access information resources. The organizational context variables are modifiable and therefore could be the focus of work environment interventions designed to increase nurses’ research utilization. Future research involving technology innovation needs to attend to nurses’ work environment to create a context that is supportive of change.

## Competing interests

The authors declare that they have no competing interests.

## Authors’ contributions

DD conceptualized the study, led the team to secure funding, was the Principal Investigator and is the lead author of the paper. RBH and DJ conducted the data collection and analysis regarding Best Evidence For Nursing Plus and contributed to revising the paper. CE enabled use of the ACT measure, provided consultation regarding its scoring, and contributed to revising the paper. AK, AD, IB and LMH participated in conceptualization of the study. IB also facilitated access to Best Practice Guidelines formatted for use on handheld devices. ML and YQB conducted the data analysis. JC managed the data collection and assisted with preparing the manuscript. All authors read and approved the final manuscript.
